# Integrative Pathway-Based Approach for Genome-Wide Association Studies: Identification of New Pathways for Rheumatoid Arthritis and Type 1 Diabetes

**DOI:** 10.1371/journal.pone.0078577

**Published:** 2013-10-25

**Authors:** Finja Büchel, Florian Mittag, Clemens Wrzodek, Andreas Zell, Thomas Gasser, Manu Sharma

**Affiliations:** 1 Center for Bioinformatics Tuebingen (ZBIT), University of Tuebingen, Tübingen, Germany; 2 Department for Neurodegenerative Diseases, Hertie Institute for Clinical Brain Research, University of Tübingen, and DZNE, German Centre for Neurodegenerative Diseases, Tübingen, Germany; 3 Institute for Clinical Epidemiology and Applied Biometry, University of Tübingen, Tübingen, Germany; INSERM, France

## Abstract

Genome-wide association studies (GWAS) led to the identification of numerous novel loci for a number of complex diseases. Pathway-based approaches using genotypic data provide tangible leads which cannot be identified by single marker approaches as implemented in GWAS. The available pathway analysis approaches mainly differ in the employed databases and in the applied statistics for determining the significance of the associated disease markers.

So far, pathway-based approaches using GWAS data failed to consider the overlapping of genes among different pathways or the influence of protein–interactions. We performed a multistage integrative pathway (MIP) analysis on three common diseases - Crohn's disease (CD), rheumatoid arthritis (RA) and type 1 diabetes (T1D) - incorporating genotypic, pathway, protein- and domain-interaction data to identify novel associations between these diseases and pathways. Additionally, we assessed the sensitivity of our method by studying the influence of the most significant SNPs on the pathway analysis by removing those and comparing the corresponding pathway analysis results. Apart from confirming many previously published associations between pathways and RA, CD and T1D, our MIP approach was able to identify three new associations between disease phenotypes and pathways. This includes a relation between the influenza-A pathway and RA, as well as a relation between T1D and the phagosome and toxoplasmosis pathways. These results provide new leads to understand the molecular underpinnings of these diseases.

The developed software herein used is available at http://www.cogsys.cs.uni-tuebingen.de/software/GWASPathwayIdentifier/index.htm.

## Introduction

GWAS typically focus on single marker statistics to obtain top hits [1]. This approach led to the identification of new candidate regions/SNPs in multiple disorders. In the National Human Genome Research Institute catalogue of September 2012, only 4,392 out of 8,965 studies reported a *p*-value smaller than 10^−8^, a common statistical threshold required for genome-wide significance. This argues in favor of applying new methodologies to unravel the complex architecture of common diseases [2]. It is conceivable that SNPs, which are genuinely associated with a phenotype, may not be identified with a single GWAS due to the small effect size of risk variants. Therefore, it is plausible to hypothesize that the pathway-based approaches which jointly consider multiple SNPs or genes in the same pathway may offer an alternative approach to standard single marker statistics approaches to uncover the genetics of complex diseases. Pathway-based studies are preformed because risk alleles for any given phenotype are more likely to be distributed among certain groups of genes whose functionalities are closely related [3].

A great number of studies highlighted the usefulness of pathway-based approaches to detect new genes/SNPs which otherwise would be skipped due to strict statistical stringencies, which are usually applied in GWAS to avoid false positive findings [4], [5]. For example, a study by Wang *et al*., which used Wellcome Trust Case-Control Consortium (WTCCC) GWAS data on Crohn's disease (CD), highlighted the role of numerous genes involved in the IL12/IL23 pathway, which were only identified through meta-analyses of several GWAS [6].

So far, most of the pathway studies based on GWAS data published to date have used only genotypic data or summary statistics to identify new pathways for different diseases. These approaches use different algorithms and database and they led to the identification of novel disease associated pathways for diverse complex phenotypes (PoDA [7], KGG [8], IPA and GSEA [9], ICSNPathway [10], BGSAsnp [11], WGNCA [12], GenGen [6], GRAIL [13]. There are only few published methods that also consider the importance of protein interaction data and automatically account for interactions that are not stored in the employed pathway databases. Path, for example, allows the user to integrate his own knowledge about interacting SNPs [14] to improve the detection of associated pathways; Baranzini et al. used an application which identifies sub-pathways using protein interactions [4]; DAPPLE is a tool that inverts the traditional approaches by building disease associated protein interaction networks, using interaction information from pathway databases [15]; Lastly, GenGen combines single SNP statistics of overlapping genes [6] in order to detect associations of SNPs and pathways. A more detailed overview of the mentioned pathway approaches, the investigated diseases and applied databases is shown in Table S1. Nevertheless, all these above mentioned studies did not investigate the role and influence of the most significant SNPs on the pathway analysis results.

Therefore, we performed a multistage pathway analysis, which allows the combination of GWAS data with pathway and protein-interaction information to reveal novel pathways. The applied protein-interaction data consists of both known and predicted interactions from published prediction algorithms at the domain level [16]. The advantage of using predicted protein-interactions with genotypic data is to augment common pathway knowledge with new possible interactions. This information helps to identify new pathways involved in the disease pathogenesis. Additionally, we developed a stand-alone user-friendly Java application, named GWAS Pathway Identifier, to perform this multistage pathway analysis for complex diseases.

## Results

### Evaluation using GWAS Pathway Identifier

All 255 pathways from the KEGG database have been included in our study. A total of 361,963 SNPs for CD, 362,229 SNPs for RA, and 362,548 SNPs for T1D encompassing 16,820 genes were included in the study. We were able to identify 157 pathways for CD, 56 for RA and 46 pathways for T1D with a *p*-value smaller than 10^−3^ (see Tables S2, S3, and S4). Among these pathways, we identified the influenza-A pathway for RA as well as the phagosome and toxoplasmosis pathway for T1D, which represent as-yet unknown genotypic links to the disease phenotype (see Table S5). These three pathways were only discovered by using the integrative pathway approach in our analysis. This highlights the usefulness of integrating protein-interaction databases in GWAS settings to uncover new leads that decipher the genetic etiology of complex phenotypes. Notably, using the multistage integrative pathway (MIP) approach for analyzing CD, T1D and RA, our results, in addition to identifying new pathways for RA and TID, are also in agreement with previously published studies (see Table S5).

Besides the comparison to previously published studies, we also performed an analysis using the application GenGen, a gene-set enrichment analysis [6], on all three WTCCC datasets and compared the results to our study. In contrast to GenGen, we discovered the above mentioned three new significant links to pathways associated with RA and T1D, which have not been identified by GenGen (see Table S5).

### Evaluating the bias of the pathway analysis

Using the MIP approach, we performed multiple analyses to understand the influence of significant SNPs on the best pathways for the given phenotypes. Furthermore, as described in the methods section, we evaluated the influence of the most significant markers on the pathway sets in our study by excluding markers whose *p*-values were smaller than a defined threshold. We discovered 24 pathways for CD, 31 for RA and 24 for T1D, which remained significant over all sensitivity runs (see Table 1 and Table S6). After excluding the most significant SNPs, we were not able to rediscover previously published associations to pathways that are associated with RA, T1D and CD, for instance the cytokine-cytokine pathway for CD [17].

**Table 1:** Consistently significant pathways.

Pathway	*p*-value		
	Crohn's disease	Rheumatoid arthritis	Type 1 diabetes
**Allograft rejection**	-	≤1.0E-06	≤1.0E-06
**Antigen processing and presentation**	1.6E-03	≤1.0E-06	≤1.0E-06
**Apoptosis**	-	3.0E-03	-
**Asthma**	-	≤1.0E-06	9.6E-03
**Autoimmune thyroid disease**	-	≤1.0E-06	≤1.0E-06
**Axon guidance**	-	1.6E-02	-
**B cell receptor signaling pathway**	≤1.0E-06	-	-
**Calcium signaling pathway**	-	7.2E-03	-
**Cell adhesion molecules (CAMs)**	-	≤1.0E-06	-
**Cytokine-cytokine receptor interaction**	-	1.6 E-03	-
**Endocytosis**	≤1.0E-06	2.2 E-03	-
**Epstein-Barr virus infection**	≤1.0E-06	≤1.0E-06	≤1.0E-06
**Fc epsilon RI signaling pathway**	≤1.0E-06	-	-
**Fc gamma R-mediated phagocytosis**	≤1.0E-06	-	-
**Galactose metabolism**	-	-	9.4E-03
**Glycerolipid metabolism**	3.0E-03	-	-
**Glycerophospholipid metabolism**	2.0E-04	-	-
**Graft-versus-host disease**	4.4E-03	≤1.0E-06	≤1.0E-06
**Herpes simplex infection**	1.0E-03	≤1.0E-06	≤1.0E-06
**HTLV-I infection**	≤1.0E-06	≤1.0E-06	≤1.0E-06
**Influenza A**	2.6E-03	2.0E-04	1.5E-02
**Inositol phosphate metabolism**	3.4E-03	-	-
**Intestinal immune network for IgA production**	-	≤1.0E-06	1.5E-02
**Jak-STAT signaling pathway**	≤1.0E-06	-	-
**Leishmaniasis**	1.6E-03	8.0E-04	1.2E-02
**Leukocyte transendothelial migration**	-	1.6E-02	-
**MAPK signaling pathway**	-	3.4E-02	-
**Measles**	-	1.0E-03	-
**Natural killer cell mediated cytotoxicity**	-	2.0E-04	9.2E-03
**Neuroactive ligand-receptor interaction**	≤1.0E-06	-	-
**Non-small cell lung cancer**	≤1.0E-06	-	-
**Pathways in cancer**	≤1.0E-06	-	-
**Phagosome**	-	≤1.0E-06	2.5E-02
**Phosphatidylinositol signaling system**	6.0E-04	-	-
**Rheumatoid arthritis**	5.0E-03	3.2E-03	8.4E-03
**RNA transport**	-	-	8.2E-03
**Shigellosis**	-	-	2.4E-02
**Small cell lung cancer**	≤1.0E-06	-	-
**Staphylococcus aureus infection**	2.0E-04	≤1.0E-06	3.6E-03
**Systemic lupus erythematosus**	-	≤1.0E-06	2.2E-02
**Tight junction**	-	2.2E-03	-
**Toxoplasmosis**	-	1.6E-03	8.0E-03
**Tuberculosis**	4.0E-04	4.0E-04	1.2E-03
**Tumor viruses and cancer**	-	≤1.0E-06	≤1.0E-06
**Type I diabetes mellitus**	8.0E-04	≤1.0E-06	≤1.0E-06
**Viral myocarditis**	-	≤1.0E-06	≤1.0E-06

To avoid that a pathway is only significant due to a small number of significant SNPs, we performed the multistage integrative pathway (MIP) analysis pipeline four times with different constraints. In the first MIP run, all SNPs are included. In the next three runs, only those having a *p*-value smaller than a threshold of 10^−3^, 10^−4^, and 10^−5^, respectively, are included. This table lists all pathways that had a *p*-value smaller than 0.05 during all four MIP runs. The complete results of each disease are shown in supplementary Tables S2, S3 and S4, and a more detailed overview of all four MIP runs which also includes a comparison to the literature is depicted in Table S5.

### Rheumatoid arthritis

The majority of pathways that are significant in our analyses for RA are related to the immune system. Our results for RA are in agreement with previously published studies [4], [5]. We also identified the influenza-A pathway, a novel disease associated pathway. This pathway remained significant even after removing the most significant SNPs and the association has not been detected in previously published studies (see Table S5, RA).

### Type 1 diabetes

The majority of pathways that are significant in our analyses for T1D are related to immune functions. The investigation of T1D with the MIP approach and subsequent sensitivity analysis identified a relation to 24 pathways, out of which two have not been published before. The two newly identified pathways are phagosome and toxoplasmosis (see Table S5, T1D). Both pathways are directly involved in the immune defense. Therefore, a connection between T1D and autoimmune diseases is conceivable. Especially, anti-toxoplasma antibodies might have an effect to autoimmune diseases [18].

### Crohn's disease

Using the MIP approach and applying a sensitivity analysis, 24 out of 157 best pathway sets from the CD investigation remained significant (15.3%). The top hit pathways encompass mainly disease, infection and immune response pathways, such as Epstein-Barr virus infection, B cell receptor signaling and the antigen processing and presentation pathway. These findings support the theory of a connection between CD and microbe interactions [19].

Furthermore, our study clearly confirms the role of previously published pathways as important pathways in influencing the susceptibility to CD (see Table S5, CD).

### Suggested common biological mechanisms in three different phenotypes

To understand the role of common biological pathways in different phenotypes, we mined three phenotypes using SNP sets that are based on known and predicted protein-interactions and pathway information (see the methods section for more details). We observed a substantial overlap of eleven pathways between CD, RA and T1D (see Table 1). These pathways cover a broad range of different functions, such as immune responses, and provide new insights into disease-overlapping aspects. The observation of common pathways might suggest a common biological mechanism that triggers different disease phenotypes.

## Discussion

We here present the multistage integrative pathway analysis (MIP) that incorporates information from protein-interaction databases and GWAS genotypic data to perform a pathway-based analysis of GWAS. Using our MIP approach, apart from confirming previously published pathways, we also identified three pathways associated with RA and T1D. The identified pathways are considered to be associated to these phenotypes (see Table S5), even though none of the SNPs in these pathways have been identified as putative risk factor in recently published GWAS.

Additionally, we showed that it is important to consider the influence of the top significant SNPs on a pathway analysis. These SNPs distort the statistical evaluation in a manner that a whole pathway is discovered to be associated to a disease although it is only one or few SNPs which are associated to it. We overcome this problem by excluding SNPs having a *p*-value smaller than 10^−3^, 10^−4^ or 10^−5^ and performed our analysis again. Finally, we compare these results to the original study that includes all SNPs. After this comparison 24 pathways of the CD analysis, 31 pathways of the RA analysis and 24 pathways of the T1D pathways kept significant in all analysis runs. Thus, our method is not biased by single SNPs having very low *p*-values.

Up to now, there exist several pathway analysis approaches for GWAS. They differ in statistics, machine learning methods or text mining approaches which are applied for the identification of disease related pathways. Besides these fundamental differences, the used bioinformatics databases and/or the releases also vary between the studies (see Table S1). Due to these facts, it is not straightforward to compare different pathway analysis methods [20].

In this study, we used experimentally validated and predicted protein interaction data to extend the existing biological knowledge for the identification of disease associated pathways. Other approaches used such information in a different way. For instance, the application DAPPLE extracts existing interaction knowledge from pathway databases to build its own protein interaction networks [15] and the application Path requires manually entered interaction data from the user [14]. In contrast to these approaches, we automatically combine pathway data with additional protein interaction. To our knowledge there exists no similar approach which makes a direct comparison between the study results difficult. However, it was possible to compare our results to the application GenGen as this tool used similar data sources (see Table S7), to the results of [4]–[6], [21] and we additionally mined the literature to validate the presented results (see Table S5). Finally, most of our results were found by other pathways tools and in literature, thus, we only denoted those pathways as new associated disease pathways which cannot be identified by any other source.

**Figure 1:**
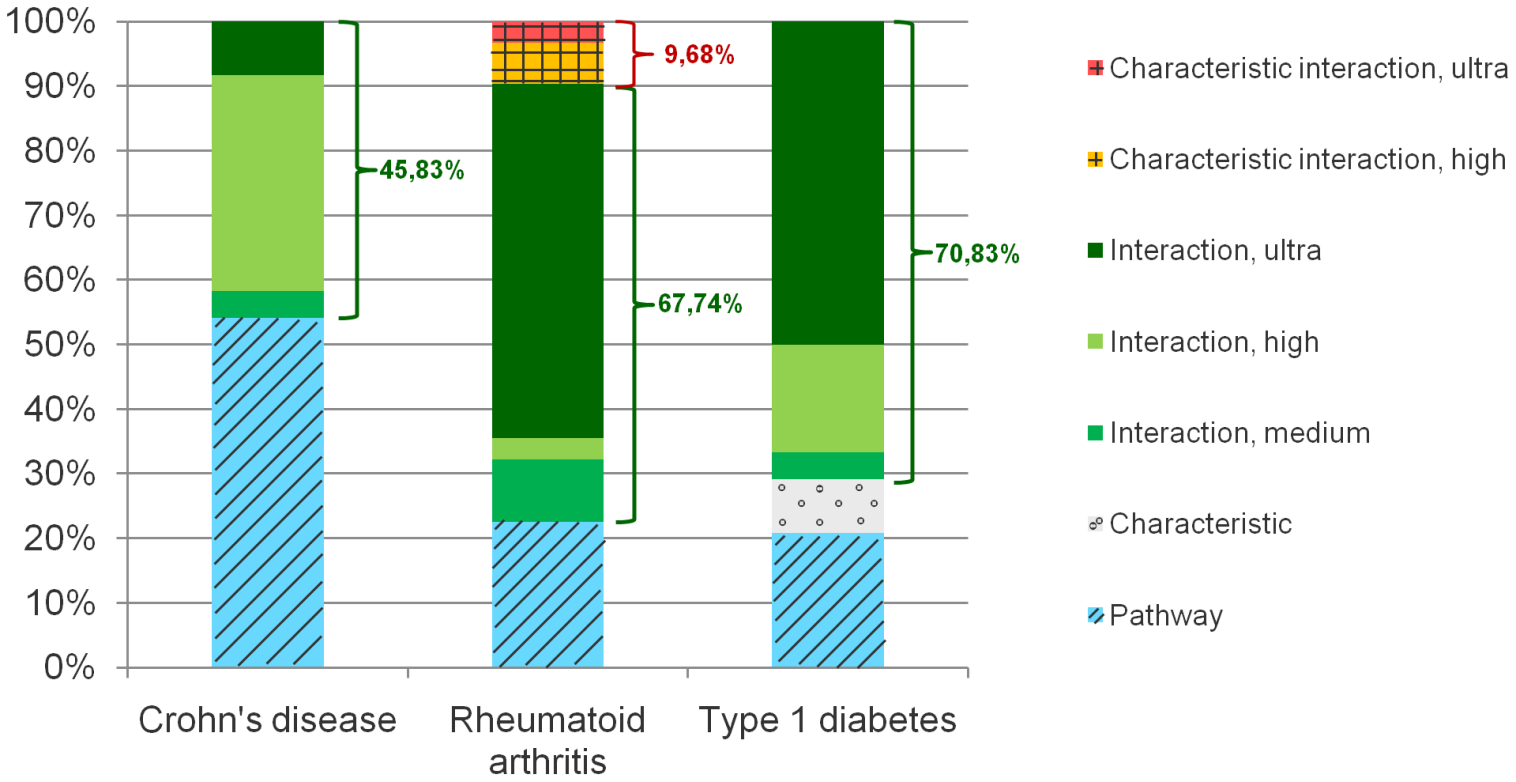
Consistency of pathway sets generated with the proposed analysis methods. The procedure described in this manuscript includes multiple analysis methods to identify significant pathways that are related to the phenotype of a given GWAS. This diagram shows with which analysis methods the consistent pathway sets that are listed in Table 2 have been determined. In average, 61% of the sets are determined with the interaction methods. In contrast, the characteristic interaction methods only identified less significant pathway sets. Concluding, it is more important to focus on the interaction based methods for the identification of important SNP sets.

Further, it was possible to show that including biological *a priori* knowledge improves the quality of SNP sets and leads to more significant results. We designed four different kinds of pathway analysis methods to build pathway-based SNP sets. Two of these methods include protein-interaction data. In summary, 45.83% for CD, 77.42% for RA, and 70.83% for T1D of the best pathway sets are determined using the interaction methods (see Figure 1).

The majority of pathways that are significant for CD, RA and T1D cover a wide variety of different functions. For example, for CD we were able to identify pathways dealing with cell signaling (see Table 1). This includes among others, the Jak-STAT and B cell receptor signaling pathway in CD pathogenesis. The latter was already reported in other studies [22].

Likewise for RA and T1D, the majority of pathways that are top hits in our study are related to immunological functions. The involvement of immunological pathways in these disorders is not surprising and has been shown in previous studies [19], [23], [24]. The involvement of the influenza pathway in RA, however, has not been reported before and may provide new clues to understand the pathophysiology mechanism of the disease. Indeed, a recent study showed that RA patients have an increased risk of infection although the increased susceptibility to infections could not be attributed to a compromised humoral immune response [25].

The significance of the phagosome pathway in T1D seems to be obvious since it plays an important role in the immune system, whose activity is increased in T1D patients. The pathways identified in RA and T1D have not been nominated by other pathway studies.

The identification of common pathways for different phenotypes suggests common molecular underpinnings for these disorders which is likely due to a cumulative effect of multiple low risk factors in these pathways that might trigger different phenotypes. For example, the allograft rejection and the intestinal immune network for IgA production pathways have been shown to be involved in RA and T1D [26]–[29].

Out of many publicly available databases such as BioCarta and Gene Ontology (GO), we choose to construct our pathways based on KEGG PATHWAY. Each of these databases has its own advantage and disadvantage. However, we chose KEGG, because its pathways are manually curated, represent a high-quality resource and provides a well-defined amount of metabolic and signaling pathways [30], [31]. In contrast, GO is an ontology and has the purpose of categorizing biological terms [32] while KEGG aims at reflecting biological workflows.

Our study also has a few limitations. Despite the use of an integrative approach in deciphering newly associated pathways for diverse phenotypes for any given pathway study, the basic unit of analysis is a pathway, which is extracted from existing databases. Despite the advancement in genomics, the function of many genes is not deciphered and hence those genes cannot be assigned to pathways. Moreover, recent studies also suggest the role of non-coding regions in influencing the susceptibility to complex phenotypes, therefore, like GWAS, pathways also capture a relatively modest amount of genetic variations. The technological advancement will expedite the annotation of the human genome, which will help to curate comprehensive pathway data sets for genetic studies.

## Materials and Methods

### Study cohorts

We used four publically available Wellcome Case-Control Consortium (WTCCC) GWAS datasets for our study: Crohn's disease (CD), rheumatoid arthritis (RA) and type 1 diabetes (T1D) as cases, and the British Birth cohort as control. These datasets have already been subject to extensive quality control procedures, whose details are described elsewhere [33]. Additionally, we filtered all datasets to exclude samples or SNPs with more than 5% missing values, variants with less than 5% minor allele frequency, and samples deviating from the Hardy-Weinberg equilibrium using the PLINK command line tool [34]. A detailed overview of the datasets is shown in Table 2.

**Table 2:** Overview of the investigated WTCCC data sets.

WTCCC data set	Number of cases	Number of controls	Number of SNPs after quality control			
			All	*p*-value <1.0^−05^	*p*-value <1.0^−04^	*p*-value <1.0^−03^
**Crohn's disease**	2,005	1,504	361,963	361,872	361,637	360,543
**Rheumatoid arthritis**	1,999	1,504	362,229	361,996	361,866	361,240
**Type 1 diabetes**	2,000	1,504	362,548	362,153	361,981	361,348

The SNPs were genotyped with an Affymetrix GeneChip 500K. The 1958 British Birth cohort with 1,504 samples was used as control.

### Overview of the study

**Figure 2:**
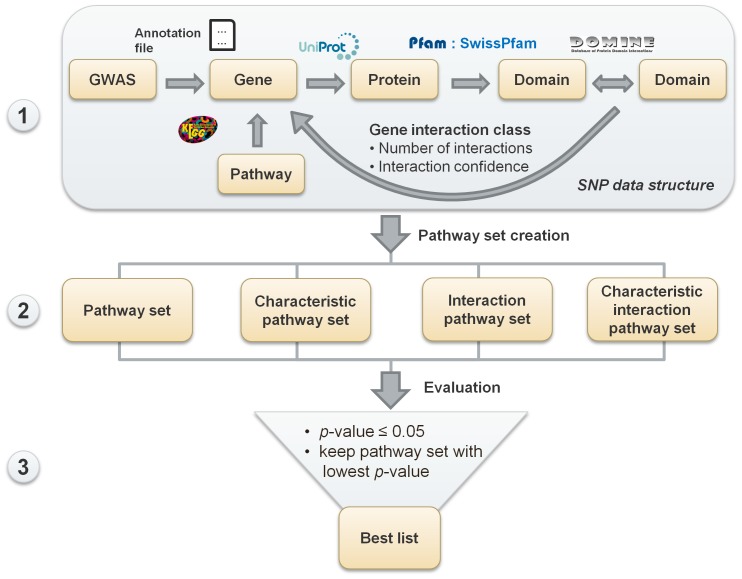
Established analysis pipeline for a multistage integrative pathway analysis. The analysis consists of three steps: 1) Construction of the SNP data structure, 2) Creation of the pathway sets, and 3) evaluation of these sets and determination of the best ones. The data structure is built by mapping all SNPs to their corresponding gene. Depending on the domain-interactions of the gene's proteins, each gene is assigned to a gene interaction class, which describes the number of interactions and the interaction confidence of the encoded proteins. For this purpose, information from UniProt, Pfam, DOMINE, and KEGG is used. Additionally, the KEGG pathways of the genes are determined. In the second step, four different pathway sets are built (for more details see Figure 3). In the final step, these sets are statistically evaluated with a variation of the Fisher's test statistic. Since there are several pathway sets built for one pathway, a best list is determined containing exclusively one set per pathway which has a *p*-value smaller or equal 0.05.

We developed a three step multistage integrative pathway (MIP) analysis pipeline to perform a pathway-based GWAS analysis for each WTCCC dataset. In the first step, we constructed a multi-layered data structure consisting of SNP, gene, known and predicted protein-interaction, and pathway data. Based on this data structure, four different kinds of SNP sets were generated in the second step. Finally, these sets were evaluated with a modification of the Fisher's combined statistic approach using 5000 permutations and a best list of the pathways was determined (see Figure 2 and a more detailed method overview in Figure S1).

The MIP was performed four times: the first time with all SNPs and three times with an exclusion of the most significant SNPs having a *p*-value smaller than a predefined threshold. For these three runs, we selected a threshold of 10^−3^, 10^−4^, and 10^−5^ (see Table 2 for detailed SNP numbers). With the exclusion of the top significant marker hits, we avoid that a pathway is significant only due to a small number of markers with extraordinary significance. Finally, only those pathway sets are selected which have a *p*-value smaller than 0.05 in all four MIP runs. The results are summarized in Table 1 and a detailed view is given in Table S6.

In addition to our study, we performed an analysis for the CD, RA and T1D dataset with the program GenGen using the published GenGen pathway-definition file for KEGG pathways (kegg.gmt) [6]. Finally, we compared the top pathways of our study to the results from GenGen, to the pathway analysis approaches from [4], [5], [21], [35], and to previously published literature (see Table S5).

### SNP data structure

We designed a special data structure for this analysis by incorporating information content from KEGG PATHWAY (release 63.0, July 1, 2012 [36]), as well as known and predicted protein-interaction data from the databases UniProt (release 2011_02 [37]), Pfam (release 25, March 2011 [38]) and DOMINE (v2.0, September 2010 [16]). Our data structure implemented in GWAS Pathway Identifier consists of five connected layers: (i) the **SNP layer** consists of SNPs and *p*-values determined using a single marker analysis of PLINK [34]; (ii) the **pathway layer** contains pathways from KEGG; (iii) the **gene layer** provides genes for the SNPs, a gene interaction class (see below), and a connection to the pathway and protein layer; (iv) the **protein layer** combines data from UniProt and Pfam and provides all encoded proteins of the gene layer and protein domains; (v) the **domain-interaction layer** contains known and predicted domain-interactions from the DOMINE database (see Figure 2).

We used the freely available mapping file from the chip manufacturer to obtain a link between SNPs and genes. Therefore, we combined two SNP annotation files as given by Affymetrix for the GeneChip 500k, Mapping250K_Sty.na32.annot.txt and Mapping250K_Sty.na32.annot.txt (both version number 32). The combined file provides NCBI gene and reference sequence identifiers to map the SNPs onto genes. With this mapping method, it was possible to assign 98.7% of the SNPs to genes. After mapping the SNPs to genes using the recommended Affymetrix annotation, 37% of these SNPs are directly located in the coding region of their associated genes and 61.7% of these SNPs are located 5′ upstream or 3′ downstream of associated genes. Those SNPs are also included in the analysis.

Depending on the domain interactions, we assigned an interaction class to each gene which reflects the number of interactions of the corresponding protein, and the interaction confidence of these interactions. We chose four interaction classes defined by the DOMINE database (ordered in descending interaction confidence): experimentally validated (EV), high interaction confidence (HC), medium interaction confidence (MC) and low interaction confidence (LC). The interaction class corresponds to the median of all interaction categories, i.e., at least 50% of the interactions must belong to the defined class or to a class of higher confidence.

The advantage of constructing such a data structure is that SNP sets can be built that reflect the biological background and biochemical interplay of the SNP genes more precisely than randomly created SNP sets.

### Construction of pathway sets

In the following, a pathway set describes a set of SNPs that meets different requirements. Thus, the basis of every set is a pathway, i.e., it consists exclusively of SNPs located on genes that are contained in this specific pathway. For our analysis, we built four different pathway sets: (i) simple pathway sets, (ii) characteristic pathway sets (iii) interaction pathway sets, and (iv) characteristic interaction pathway sets. The simplest set is the **pathway set**, which contains all SNPs located in any gene of a specific pathway. In contrast the **characteristic pathway set** contains only those SNPs of genes occurring exclusively in the pathway. If a gene occurs in more than one pathway it is not considered for the analysis. In addition to that, the characteristic pathway set allows us to explore the influence of overlapping genes between different pathways.

**Figure 3:**
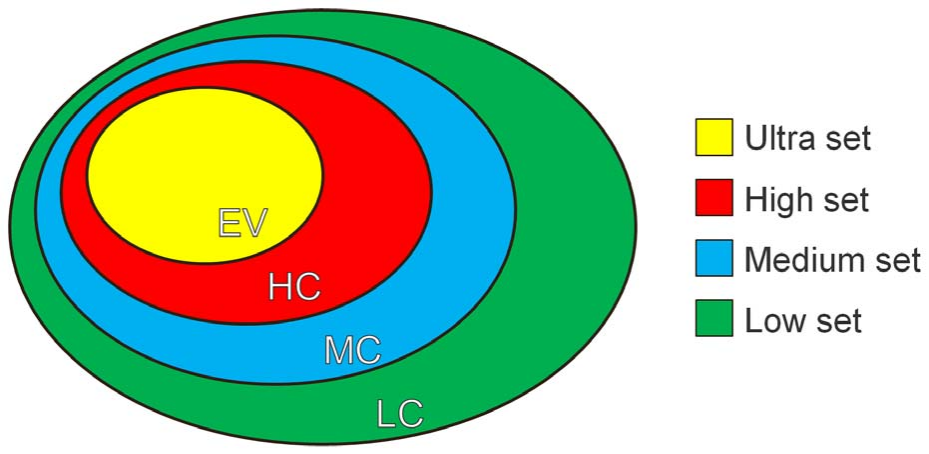
Definition of the interaction pathway sets. We built four interaction pathway sets: ultra (yellow), high (red), medium (blue) and low (green) depending on the interaction classes of the genes. These interaction classes are either EV (experimentally validated), HC (high interaction confidence), MC (medium interaction confidence) and LC (low interaction confidence). The low interaction set is the superset of all interaction sets because it includes genes of all interaction classes. In contrast, the smallest ultra set only contains the genes of the EV class.

**Figure 4:**
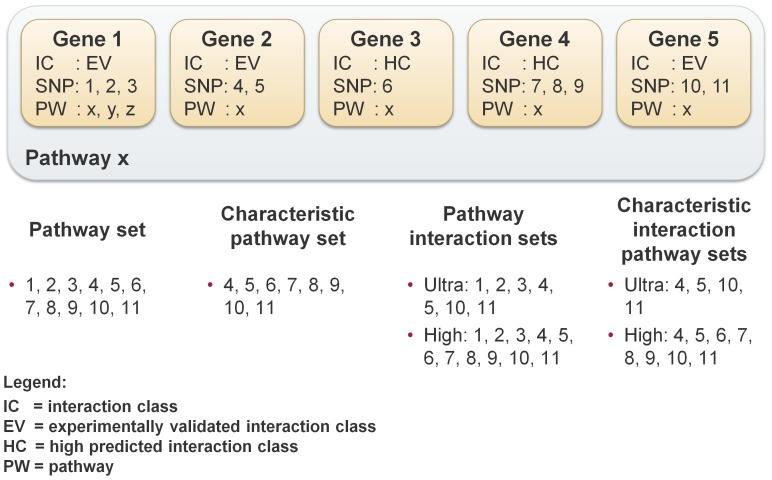
Pathway set creation example. This example shows how the different pathway sets are built for a given pathway *x*. The pathway *x* is depicted as blue rectangle and the genes 1 to 5 as orange rectangles. For the pathway *x*, six different pathway sets are created. First, the **pathway set** containing the SNPs of all genes occurring in pathway *x*. Second, the **characteristic pathway set** that only contains the SNPs of those genes occurring exclusively in pathway *x*, i.e., genes 2, 3, 4 and 5. Since gene 1 also shows up in pathways *y* and *z*, the SNPs of these genes are not considered in the characteristic pathway interaction set. Third, two **pathway interaction sets** are created: first, the ultra-set for the SNPs of the genes assigned to the EV interaction class and second, the high-set with SNPs of genes assigned to the HC and EV class. Finally, the **characteristic interaction pathway sets** are generated. These sets are built similar to the interaction pathway sets but they contain only those SNPs of genes occurring exclusively in pathway *x*. In contrast to the ultra interaction set, the ultra characteristic interaction set does not include the SNPs 1, 2 and 3 because the corresponding gene also occurs in pathways *y* and *z*.

The **interaction pathway set** additionally considers the gene interaction classes for the SNP-set construction. For each pathway, four different sets are created depending on the gene interaction classes: ultra-set, high-set, medium-set and low-set. The low-set is the superset of all *interaction-pathway sets* and includes the SNPs of genes of all interaction classes. The medium-set contains only SNPs of genes assigned to the EV, HC and MC class. The high-set is built by genes with EV and HC class and finally, the ultra set consists of the SNPs of the EV class (see Figure 3). The **characteristic interaction pathway set** is a combination of the *characteristic pathway set* and the *interaction pathway set*. A separate set is built for each interaction class analogous to the interaction pathway sets. But only those SNPs are included, whose corresponding gene occurs exclusively in this pathway. This is similar to the generation of the characteristic pathway sets. An example of the construction of these pathways sets is shown in Figure 4.

### Statistical analysis of pathway sets

We applied the statistical method proposed by De la Cruz et al. with 5000 permutations to evaluate the defined pathway sets [39]. We used the algorithm with 5000 permutations because our analyses showed that more permutations did not yield any improvements. The method is a variation of Fishers's combined probability test and implicitly overcomes the problem of handling linkage disequilibrium between SNPs, multiple testing and an adjustment of different sizes of genes. Briefly, the method proceeds as follows: for each pathway set 5000 new SNP sets are defined with randomly permuted disease states. The number of SNPs in these new sets is equal to the number in the original pathway set. Then, for each set a *p*-value is calculated. Finally, it is determined how often the original pathway set performs better than the randomly permuted ones. Notably, by comparing against randomly generated data, this method already performs an FDR and calculates the *p*-value on this basis [39]. In this study, we denote the resulting performance of an analysis with this method as the “set *p*-value”.

## Supporting Information

Figure S1 **A detailed overview of the analysis pipeline for a multistage integrative pathway analysis.** In addition to Figure 2, this supplementary Figure shows a more detailed overview of the analysis pipeline. The topmost blue rectangle depicts the input files which are needed for the analysis. The input files (bed, bim and fam) contain the GWAS data and can be generated using PLINK [34]. The SNP annotation file is provided by the chip manufacturer and contains a mapping from SNPs to genes. In the second rectangle, the construction of the SNP data structure is described step-by-step and the used bioinformatics databases with the release number are shown. In the third rectangle, the construction of the different pathway sets is described and how they are evaluated. The undermost rectangle shows how the most significant pathway set is determined and merged into the best list. This list finally summarizes the most significant pathways sets which are associated to the investigated disease.
(TIF)

Table S1 **Overview of several pathway analysis studies.** This table gives an overview of related pathway analysis studies for GWAS. The methods are briefly described and the investigated disease and used bioinformatics databases are listed.
(XLSX)

Table S2 **Best lists of the MIP analyses of Crohn's disease.** This excel file contains four spreadsheets presenting the results of our MIP analysis for Crohn's disease (CD): the best list of the analysis with all SNPs, the best list of the analysis using SNPs having a *p*-value ≤10^−3^, the best list of the analysis using SNPs having a *p*-value ≤10^−4^ and the best list of the analysis using SNPs having a *p*-value ≤10^−5^.
(XLSX)

Table S3 **Best lists of the MIP analyses of rheumatoid arthritis.** This excel file contains four spreadsheets presenting the results of our MIP analysis for rheumatoid arthritis (RA): the best list of the analysis with all SNPs, the best list of the analysis using SNPs having a *p*-value ≤10^−3^, the best list of the analysis using SNPs having a *p*-value ≤10^−4^ and the best list of the analysis using SNPs having a *p*-value ≤10^−5^.
(XLSX)

Table S4 **Best lists of the MIP analyses of type 1 diabetes.** This excel file contains four spreadsheets presenting the results of our MIP analysis for type 1 diabetes (T1D): the best list of the analysis with all SNPs, the best list of the analysis using SNPs having a *p*-value ≤10^−3^, the best list of the analysis using SNPs having a *p*-value ≤10^−4^ and the best list of the analysis using SNPs having a *p*-value ≤10^−5^.
(XLSX)

Table S5 **Literature comparison.** This table presents for each investigated disease the comparison of the MIP analysis results to literature and other pathways analysis tools. The red font indicates that this pathway was found by other pathway analysis tools. No highlighting is used if the pathway-disease association has been reported previously in other literature. A yellow highlighting indicates that other literature reported the pathway-association as a side-effect and orange is used if no supporting literature could be found. Abbreviations: CD =  Crohn's disease, RA =  rheumatoid arthritis, T1D =  type 1 diabetes.
(XLSX)

Table S6 **Sensitivity analysis.** This table presents the joined results of all four MIP analyses for each disease. The pathways which kept consistently significant during all analyses in all diseases are highlighted in yellow. Abbreviations: CD =  Crohn's disease, RA =  rheumatoid arthritis, T1D =  type 1 diabetes.
(XLSX)

Table S7 **Comparison to GenGen.** This table shows a comparison of the results of MIP and GenGen. The pathways which kept significant during our sensitivity analysis are highlighted in yellow. Abbreviations: CD =  Crohn's disease, RA =  rheumatoid arthritis, T1D =  type 1 diabetes.
(XLSX)
