# Supplementary figures and images for: Integrative Pathway-Based Approach for Genome-Wide Association Studies: Identification of New Pathways for Rheumatoid Arthritis and Type 1 Diabetes
Source: PLoS One. 2013 Oct 25;8(10):e78577. doi: 10.1371/journal.pone.0078577 (PMC3808349; doi:10.1371/journal.pone.0078577)

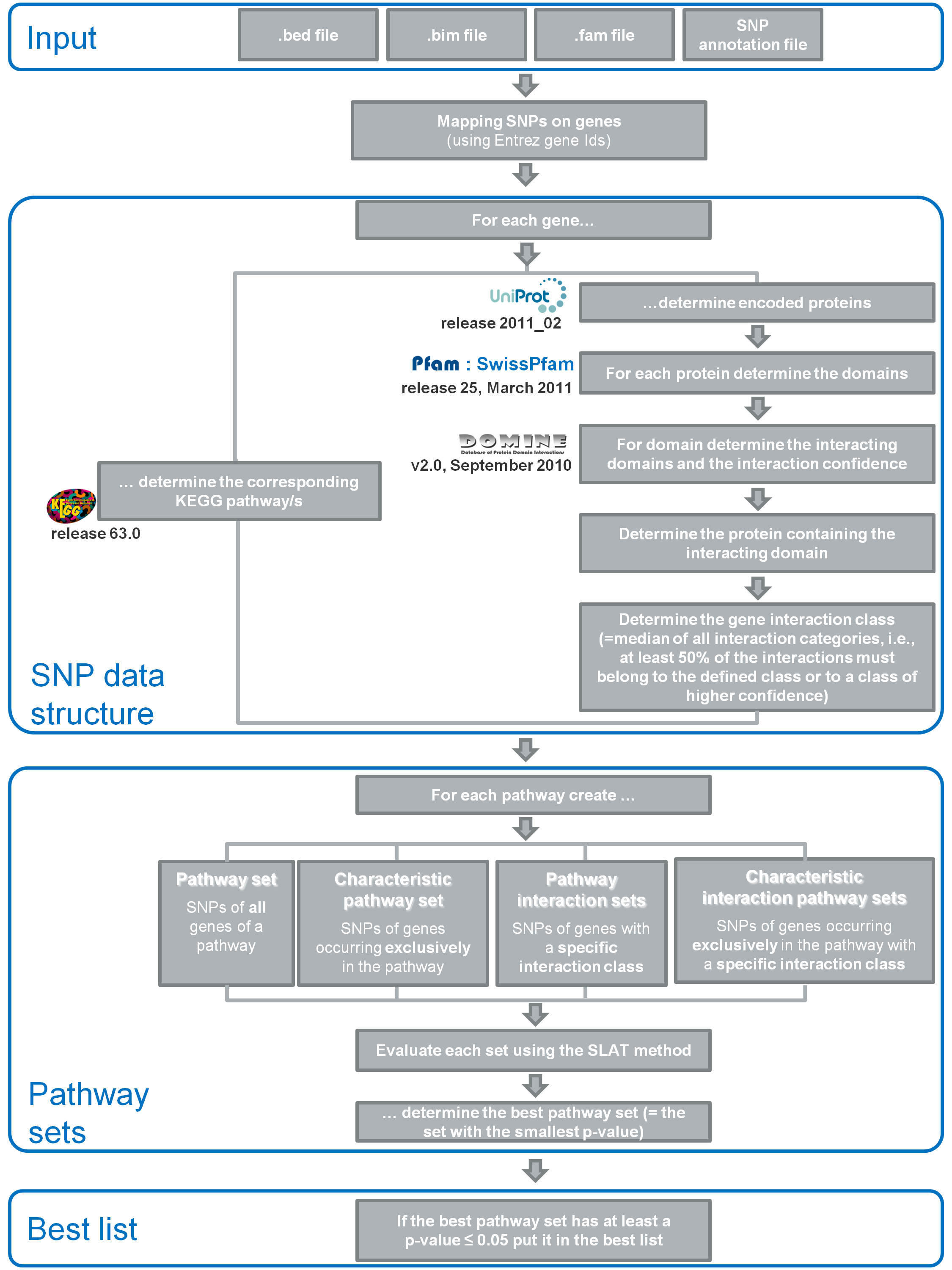

Supplement: Figure S1 — A detailed overview of the analysis pipeline for a multistage integrative pathway analysis. In addition to Figure 2, this supplementary Figure shows a more detailed overview of the analysis pipeline. The topmost blue rectangle depicts the input files which are needed for the analysis. The input files (bed, bim and fam) contain the GWAS data and can be generated using PLINK [34]. The SNP annotation file is provided by the chip manufacturer and contains a mapping from SNPs to genes. In the second rectangle, the construction of the SNP data structure is described step-by-step and the used bioinformatics databases with the release number are shown. In the third rectangle, the construction of the different pathway sets is described and how they are evaluated. The undermost rectangle shows how the most significant pathway set is determined and merged into the best list. This list finally summarizes the most significant pathways sets which are associated to the investigated disease. (TIF) [file pone.0078577.s001.tif]
